# Comparative Study on Laser Welding Thick-Walled TC4 Titanium Alloy with Flux-Cored Wire and Cable Wire

**DOI:** 10.3390/ma16041509

**Published:** 2023-02-10

**Authors:** Laibo Sun, Mingqiu Wang, Lujun Huang, Naiwen Fang, Pengbo Wu, Ruisheng Huang, Kai Xu, Xingxing Wang, Jian Qin, Shuai Li, Weimin Long

**Affiliations:** 1 Harbin Welding Institute Limited Company, Harbin 150028, China; 2 School of Materials Science and Engineering, Harbin Institute of Technology, Harbin 150001, China; 3 Shanghai Zhongxun Technology Co., Ltd., Shanghai 201200, China; 4 Henan International Joint Laboratory of High-efficiency Special Green Welding, North China University of Water Resources and Electric Power, Zhengzhou 450045, China; 5 Zhengzhou Research Institute of Mechanical Engineering Limited Company, Zhengzhou 450001, China

**Keywords:** flux-cored and cable wires, TC4 titanium alloy, laser wire filling welding, microstructure and mechanical properties

## Abstract

In the welding process of thick-walled titanium alloys, the selection of the wire type is one of the critical factors affecting the welding quality. In this paper, flux-cored and cable wires were used as filler materials in the welding of thick-walled titanium alloys. The macrostructure, microstructure, texture, and grain size of both welded joints were compared by employing an optical microscope (OM), scanning electron microscope (SEM), and transmission electron microscope (TEM), and the tensile and impact properties were also evaluated. The comparison result showed that the fusion zone microstructure of both welded joints was dominated by a basketweave structure composed of interwoven acicular α′ martensite, whereas the microstructure of flux-cored wire welded joints was finer, and the degree of anisotropy was low. The strength of both welded joints was higher than that of the base metal, ensuring that fracture occurred in the base metal area during tension. The Charpy impact energy of the flux-cored wire welded joint was 16.7% higher than that of the cable wire welded joint, indicating that the welded joint obtained with the flux-cored wire performed better in the welding process of thick-walled titanium alloys.

## 1. Introduction

TC4 titanium alloy is increasingly applied in the aerospace and defense military fields due to its high specific strength, outstanding comprehensive performance, and excellent corrosion resistance [1]. With the expansion of application areas and increased market demand, the welding requirements for TC4 titanium alloy are growing. However, because of the high activity of titanium alloys, it is highly susceptible to oxidation during the welding process, forming a gas-rich layer, which can increase the welding difficulty [2,3,4]. Additionally, the welding of thick-walled titanium alloys is prone to problems such as porosity [5], cracking [6], deformation [7,8], and failure to meet performance requirements [9].

To address these problems, researchers have proposed improvements to the weld quality of titanium alloy by optimizing the welding process or supplementing it with new techniques, such as the use of different shielding gases [10], adjusting the heat input [11], numerical simulation-assisted optimization [12], applied magnetic fields [13,14], post treatment or pretreatment [15,16,17], and ultrasonic strengthening [18]. In addition to these ways to effectively improve the weld quality, the choice of wire type can also have a significant impact on the weld quality. Bajic [19] compared the weld quality between solid and flux-cored wires. He proposed that flux-cored wire could transfer the molten metal in fine droplets, making it easier to improve the welding efficiency than with conventional solid wires. In addition, researchers found that flux-cored wires could enhance the performance of welded joints. Researchers also found that flux-cored wire also has many other advantages, such as high welding process stability, a large process window range, less oxidation, and good wettability, contributing to the trend of this type of wire gradually replacing solid metal wires [20]. Therefore, a lot of work has been carried out by researchers on the use of flux-cored wires to achieve the welding of titanium alloys. Prilutsky [21] used the arc welding method to weld titanium and titanium alloys using flux-cored wires and analyzed the characteristics of the welded joints. He suggested that flux-cored wires can cause arc shrinkage and increase current density in the anode region during the welding process, increasing the depth of the melt and effectively improving efficiency and weld quality. Bao [22] developed a new titanium alloy flux-cored wire containing TiB2, Al60-V40, and Ti-6Al-4V mixed powders, which was used for arc fusion wire deposition of TiB/Ti composite coatings to improve the hardness and wear resistance of titanium alloys. The results showed that after several chemical reactions, the wire melted in the arc region and turned into inhomogeneous droplets consisting of Ti-Al-V-B melt and undecomposed TiB2 particles. The obtained clad layer showed a uniform microstructure, a relatively low dilution ratio, and high hardness.

In addition to flux-cored wire, multi-strand twisted welding wire, known as stranded wire or cable wire, is also attracting the attention of researchers as a new type of structural welding material. Cable wire is made of multiple monofilaments spirally stranded according to a specific structure. Due to its particular physical structure, it has unique welding arc characteristics, energy distribution characteristics, and process characteristics during the welding process. Compared with traditional solid wire, cable wire has been comprehensively improved and expanded in terms of melt drop transition characteristics and melt pool flow pattern, as well as the design and manufacturing of alloy composition. It is helpful to control the welding process in terms of melt width, depth, and height, which significantly improves welding efficiency and joint performance [23,24]. The characteristics of cable wire are mainly derived from the spiral structure, which can effectively promote the rotation of the arc and stir the molten pool, enhance the fluidity of the molten metal, improve the transition behavior of the molten droplets, and balance the force state of the molten pool during the welding process to improve the stability of the weld [25]. In view of its many technical advantages, researchers have conducted a lot of research on this type of wire. Yang [26] et al. compared the microstructural characteristics and mechanical properties of stranded and single solid wire welds under the same welding parameters. The results showed that cable wires were highly efficient and exhibited satisfactory sidewall depth of fusion during the welding process. Li et al. [27] used multistranded composite wires to weld high-nitrogen austenitic stainless steels and investigated their organization and mechanical properties. The results showed that using cable wire can ensure the stability of the welding process.

As demonstrated from the abovementioned research progress, flux-cored and cable wires have shown certain advantages over solid wires in the welding process, and researchers have made progress in this area. However, a comparison between flux-cored and cable wire has not yet been reported. Therefore, in this paper, we analyze and compare the microstructure, as well as the tensile and impact properties, of TC4 titanium alloy welded joints with those of flux-cored and cable wires to provide support for the application of both wire types in the titanium alloy welding field.

## 2. Materials and Methods

### 2.1. Meterials

**Figure 1:**
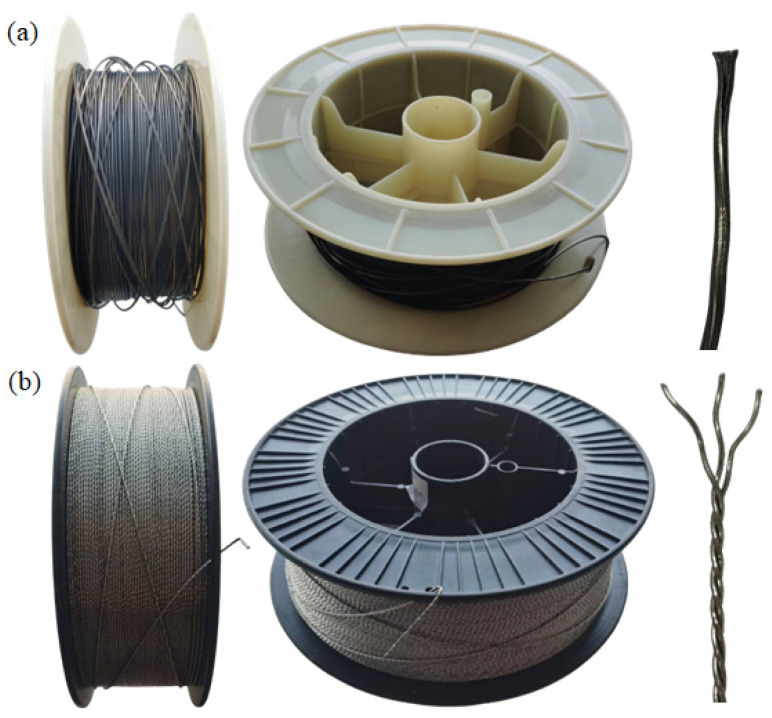
Comparison of the morphology and structure of flux-cored wire and cable wire: (**a**) flux-cored wire; (**b**) cable wire.

A Ti-6Al-4V titanium alloy plate with dimensions of 400 mm × 200 mm × 20 mm was selected for the test. Before welding, a solution of 5% HF + 30% HNO_3_ (volume fraction) was used for acid pickling of the plate to be welded to remove surface oil and oxides. The pickled plate was then washed with water and alcohol to remove the pickling solution and dried. The filler metals were 1.6 mm diameter Ti-Al-V flux-cored wire and 1.6 mm diameter TC4 cable wire. The chemical compositions of the base metal and filler wires (flux-cored and cable wires) are shown in Table 1. The morphology and characteristics of both wires are shown in Figure 1.

### 2.2. Methods

**Figure 2:**
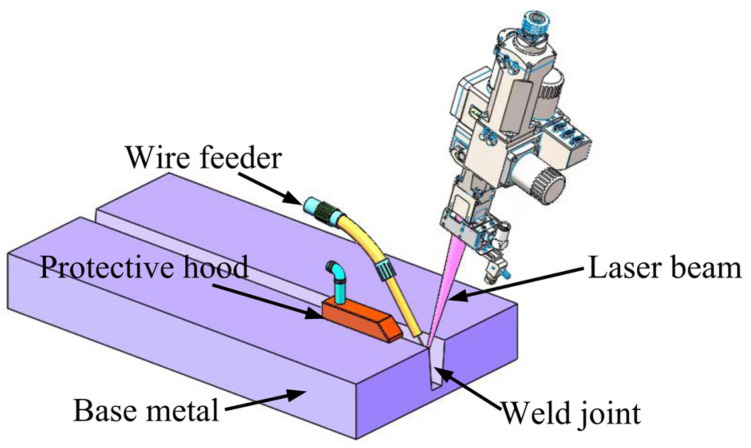
Schematic diagram of the welding process.

The plate to be welded was processed into a Y-bevel before welding, with a blunt bevel edge of 2 mm, a bevel root clearance of 3.2 mm, and a bevel angle of 1.5° on one side. Laser wire filling welding was adopted for bevel filling. An IPG YLS-6000 fiber laser was applied as a welding heat source, and Fronius KD 1500 D-11 (manufactured by Fronius International GmbH in Austria) was used as a wire feeder. They were integrated with KUKA robotics (manufactured by KUKA Roboter GmbH in Augsburg of Germany) to collaborate in controlling the welding trajectory. A schematic representation of the welding process sand the specific welding process parameters are shown in Figure 2 and Table 2, respectively.

### 2.3. Sample Preparation and Characterization

**Figure 3:**
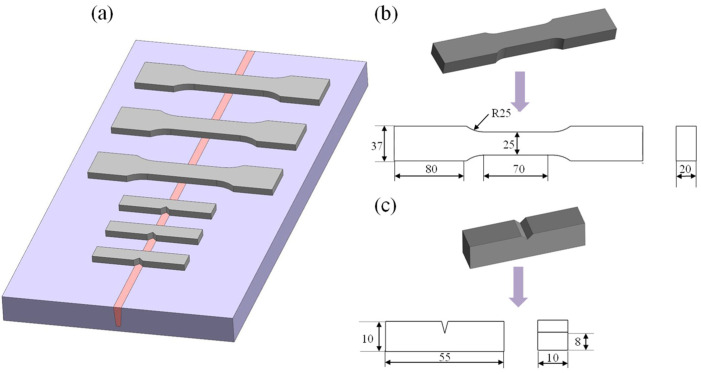
Schematic diagram of the sampling location and specimen size: (**a**) sampling location; (**b**) tensile specimen size; (**c**) impact specimen size.

After obtaining the TC4 titanium alloy welded joints, the microstructure and mechanical properties of the two joints were characterized and tested. The sampling locations and specimen sizes are shown in Figure 3. An OLYMPUS CKX53 optical metallurgical microscope was used to observe the microstructure of different regions, including the base metal (BM), heat-affected zone (HAZ), and fusion zone (FZ). EVO 18 scanning electron microscope (SEM) and electron backscatter diffraction (EBSD) were applied to observe the texture, phase composition, and grain size. The phase and microstructural morphology were characterized by a FEI Talos F200X transmission electron microscope (TEM). In addition, a room-temperature tensile test was carried out using an AG-X PLUS electron-tensile tester at a rate of 1 mm/min. A room-temperature Charpy impact test was performed using a JB-300B.

## 3. Results and Discussion

### 3.1. Microstructural Comparison

**Figure 4:**
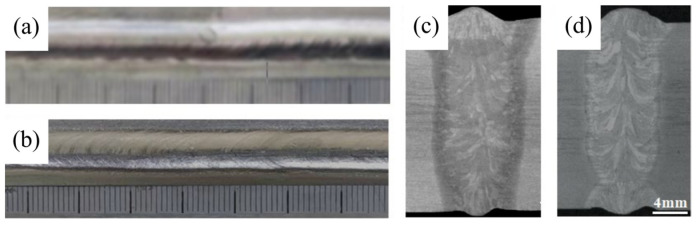
Comparison of the macromorphology of welded joints with flux-cored wire and cable wire welding joints: (**a**,**c**) flux-cored wire welding joints; (**b**,**d**) cable wire welding joints.

**Figure 5:**
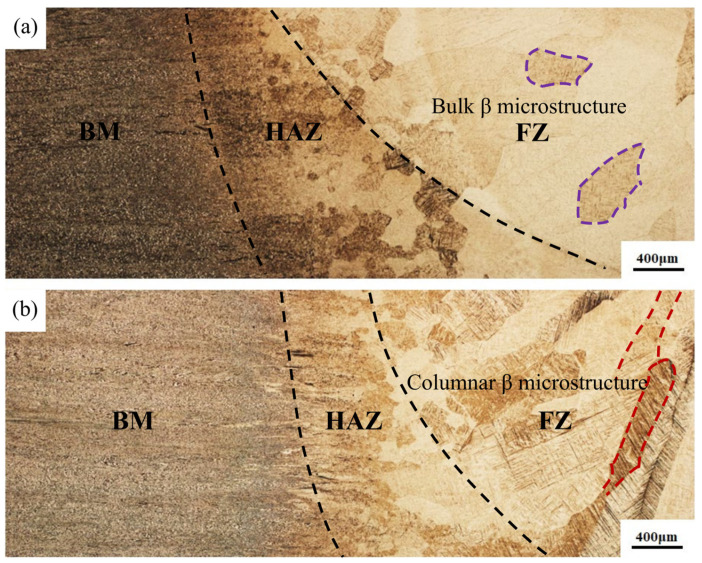
Microstructure of flux-cored wire and cable wire welding joints comparison: (**a**) flux-cored wire welding joint; (**b**) cable wire welding joint.

Figure 4 shows the overall and cross-sectional macroscopic appearance of the flux-cored wire and cable wire welded joints. No porosity, cracks, or sidewall fusion defects were observed. The macrostructure of flux-cored wire and cable wire welded joints were observed by optical microscope, as shown in Figure 5. As shown in the figure, the macrostructural variation in three typical regions (BM, HAZ, and FZ) of the two welded joints is relatively close. Approaching the center of the weld, the grains in the heat-affected zone and fusion zone grow significantly. The grains from the base metal to the fusion zone are gradually coarsened, reaching the maximum in the fusion zone. The gap in grain size between different regions is large. The boundaries between different regions are clear. This is because during the welding process, the microstructural transformation of the welded joint is mostly dependent on the initial microstructure of the base metal and the heat cycle during the welding process, including maximum heating temperature, dwelling time at high temperatures, and the cooling rate. Laser welding has a high peak heating temperature and a fast cooling rate during welding. The temperature of the fusion zone is the highest during the welding process, and the heat-affected zone close to it has the second highest temperature. The base metal zone has the lowest temperature and is least affected by the thermal effects of the welding process. The β grains in the fusion zone nucleate on the base metal surface near the fusion zone that is heated to a semi-molten state, which preferentially grows in the form of columnar dendrite along the direction of the temperature gradient to the center of the weld, forming an “epitaxial solidification”. This results in significant anisotropy of the microstructure. There is also a distinct difference in the morphology and grain size between the fusion zone and the heat-affected zone of the two joints, which is most evident in the fusion zone. Figure 5a shows that the fusion zone of flux-cored wire welded joints consists of a number of blocky polygonal β microstructures. In contrast, Figure 5b illustrates that the fusion zone of cable wire welded joints consists of many coarse, directionally distinct columnar dendrites. The microstructure of the cable wire welded joint microstructure is coarser and shows more significant anisotropy. In the heat-affected zone, the microstructure of both types of wire welded joints consists mainly of a coarse Widmanstatten structure. Compared with the flux-cored wire welded joint, the microstructure of the cable wire welded joint is also coarser. However, this difference is not as evident as the fusion zone, which is mainly related to the thermal effects of the welding process.

**Figure 6:**
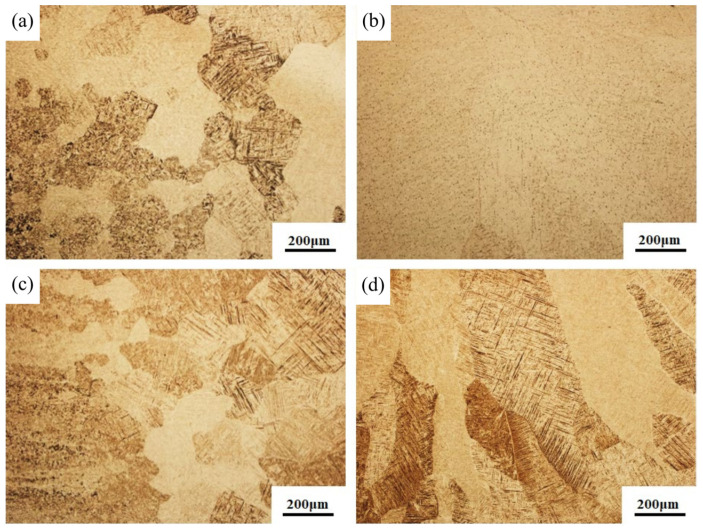
Microstructural comparison of the heat-affected zone and fusion zone: (**a**) HAZ of flux-cored wire welding joint; (**b**) FZ of flux-cored wire welding joint; (**c**) HAZ of cable wire welding joint; (**d**) FZ of cable wire welding joint.

A more detailed microstructure comparison was made between the heat-affected and fusion zones of the two types of welded joints, as observed by optical microscope. We found that the microstructure in the heat-affected zone of the flux-cored wire welded joint is composed of a small amount of primary α_p_ phase, a Widmanstatten structure, and α’ martensite. The amount of acicular α’ martensite in this region is small, and the microstructure is relatively fine. Due to the varying distance between the heat-affected zone and the melt pool, the influence also differs. The grains near the fusion line are coarser than those in the area away from the fusion line, with a larger and dense amount of acicular α’ martensite [28]. In comparison, the morphology of acicular α’ martensite in the heat-affected zone of the cable wire welded joint is more typical and has a larger aspect ratio, as shown in Figure 6a,c. This microstructure state in the heat-affected zone is mainly due to the influence of heat conduction in the fusion zone of the weld. It is known that the heat-affected zone near the weld undergoes a complete phase transformation due to a much higher temperature above the β-transformation point. There is no competitive growth mechanism for columnar dendrites in the heat-affected zone. Thus, the β phase is retained in a coarse, massive form.

**Figure 7:**
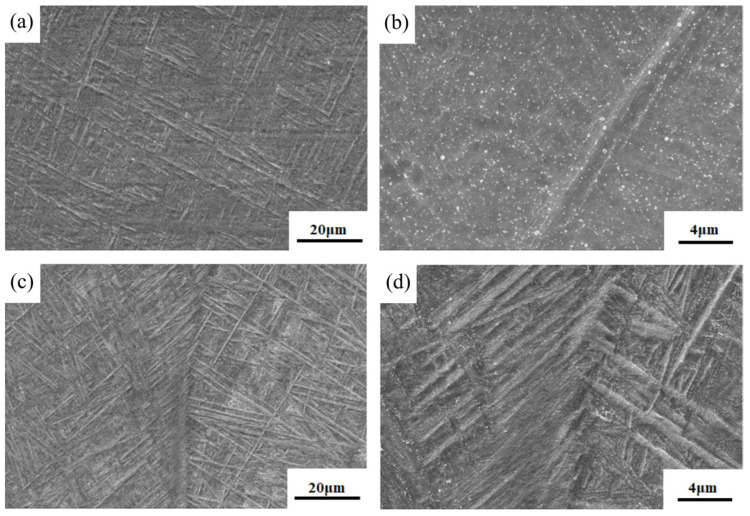
Comparison of the morphology of acicular α′ martensite between flux-cored wire and a cable wire welding joint: (**a**,**b**) flux-cored wire welding joint; (**c**,**d**) cable wire welding joint.

In the fusion zone part of both welded joints, a coarse β microstructure exists, which consists of a basketweave microstructure interwoven by a large amount of acicular α′ martensite. This microstructure occurs mainly because during the welding process, the melt pool experiences rapid melting and solidification. As a result, the alloy elements in the molten pool cannot diffuse in time during solidification, so a supersaturated α′ martensite solid solution is formed in the form of a lattice reconstruction by shear phase transition. When the joints are cooled to the solid-state phase transition temperature, the α phase prefers to nucleate in the original β-grain boundary, which contains more α stable elements. As the temperature decreases, the acicular α′ martensite phase begins to precipitate inside the β grain. During the nucleation process, the α′ martensite preferentially forms one or several parallels in the primary α′ martensite, then grows in the direction of the grain. With the nucleation and growth of the acicular α′ phase, the primary acicular martensite grows rapidly along the inside of the grain, and the growth rate becomes slower when it encounters the grain boundary. Furthermore, a fine secondary α′ phase is formed in different direction between the acicular martensite, which eventually constitutes a basketweave microstructure composed of acicular martensite [29]. However, there are distinct differences in the β-microstructural morphology under the two conditions. The acicular α′ martensite is more typical and strong in the fusion zone of the cable wire welded joint, with more evident directionality and visible grain boundaries of the adjacent β microstructure, as shown in Figure 6b,d. The two welded joints obtained under this process are close to the organization of existing laser welding of titanium alloys, but there are some differences in specific details [30].The reason for the aforementioned microstructural differences is associated the influence of different conditions of martensite nucleation. This factor may be related to e melt pool stirring during the process of cable wire welding. Its unique structural characteristics cause the stirring effect of the melt pool, so the joint is prone to form nucleation-dependent defects for martensite, providing more nucleation core. Once the martensite is nucleated, the growth is immediately completed, resulting in scattered, interlaced microstructure, as observed by SEM and shown in Figure 7.

**Figure 8:**
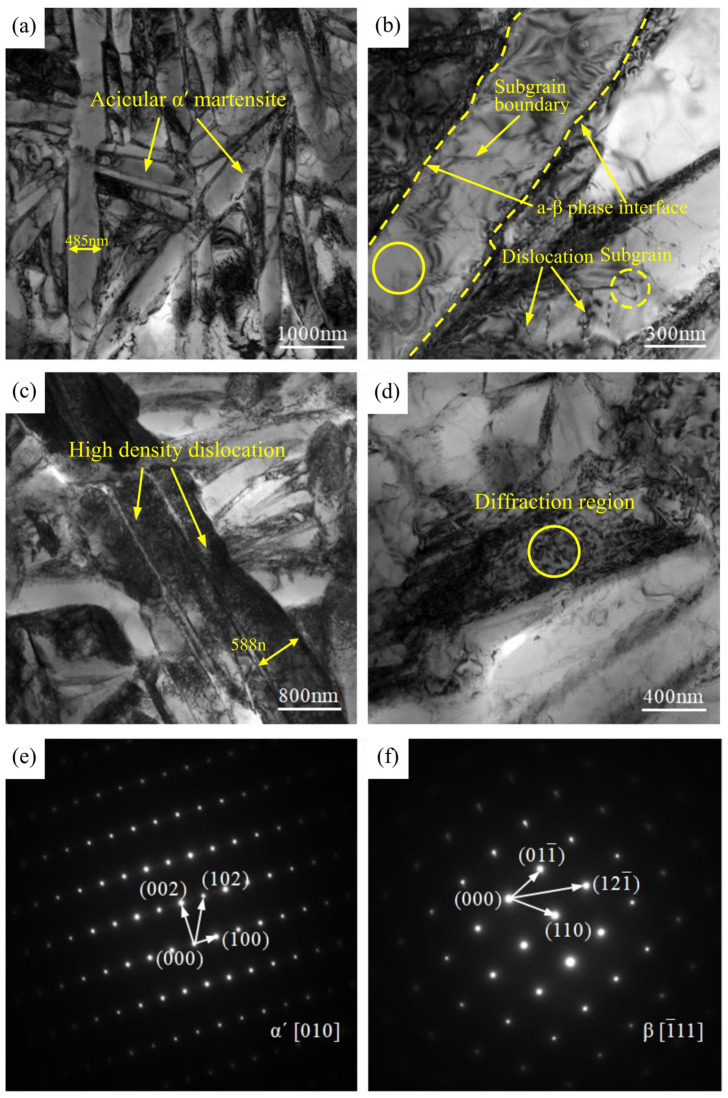
Comparison of the TEM microstructural morphology of the welding joints: (**a**,**b**) flux-cored wire; (**c**,**d**) cable wire; (**e**) diffraction spots of the α phase; (**f**) diffraction spots of the β phase.

In order to compare the microstructural characteristics of the two welded joints in depth, the fusion zone microstructure was observed using TEM. Figure 8a,b show the TEM microstructure in the fusion zone of the flux-cored wire welded joint. As seen in Figure 8a, lath α′ martensite with different growth orientations exists in this zone. During the laser welding process of TC4 titanium alloy, a β-phase transition occurs in the heating process. β grains rapidly grow and coarsen. Due to the rapid cooling of the melt pool, there is insufficient time for the grown and coarsened β phase to transform into a primary α phase, instead transforming into α′ martensite, which has the same crystal structure as the primary α phase [31,32]. The martensitic phases are parallel or staggered, with a maximum lath width of 458 nm in the field of view, and a certain amount of dislocations can be observed in some of the lath α′ martensite. Magnification of the histomorphology in the vicinity of the lath α′ martensite reveals linear dislocation lines, subgrain boundaries, and subgrains, as shown in Figure 8b. The diffraction spot in the lath position demonstrates a typical α phase, as shown in Figure 8e. Figure 8c,d shows the TEM microstructure in the fusion zone of the cable wire welded joint. Figure 8c shows some lath α′ martensite with consistent orientation, showing a coarse cluster, large and straight length, and a large aspect ratio. The maximum lath width is 588 nm in the field of view, which is significantly wider than that of the flux-cored wire welded joints. The large number of densely entangled high-density dislocations within the lath indicates that the welded joint formed under cable wire conditions completes the transition from the β phase to the non-equilibrium α′ phase at a faster rate, forming a more severe lattice distortion [25,33]. The diffraction spots at the locations marked by circles in Figure 8d are typical of the β phase, as shown in Figure 8f.

**Figure 9:**
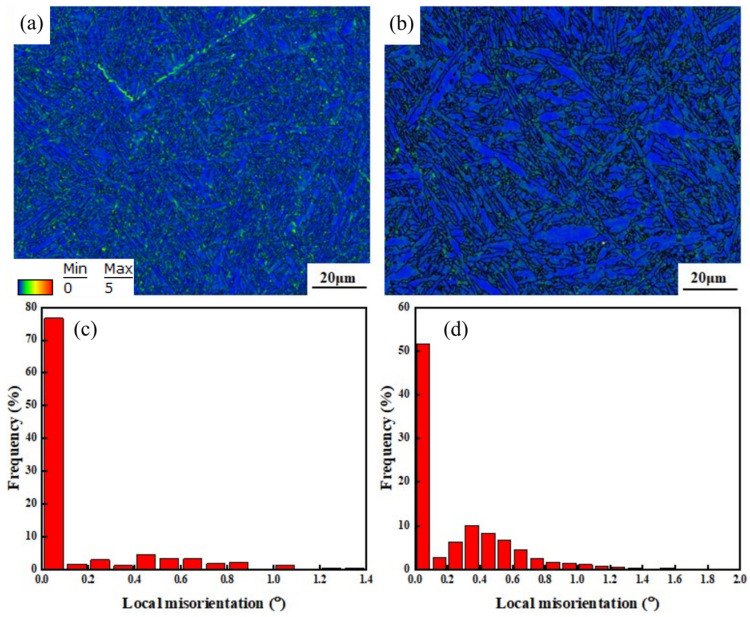
Comparison of local misorientation of the welding joints: (**a**) flux-cored wire welding joint; (**b**) cable wire welding joint; (**c**,**d**) statistical histogram for flux-cored wire and cable wire welding joints.

After an in-depth qualitative analysis of the microstructure of the two welded joints, EBSD was used to perform an accurate quantitative analysis of the microstructure and texture. As is known, KAM (kernel average misorientation) is a reflection of the average misorientation between the adjacent points, which can characterize the degree of local plastic deformation and distortion of the material. Figure 9 compares the KAM of the two welded joints, showing that both joints are dominated by the blue region representing low local misorientation. In comparison, the local misorientation of the flux-cored wire welded joint is lower, and a certain number of fine substructures exist in the microstructure, as shown in Figure 9a and as confirmed by the statistical histogram of local misorientation. In the flux-cored wire welding joint, local misorientation of less than 0.1° accounts for more than 70%, whereas in the cable welding joint, local misorientation accounts for only 50%, as shown in Figure 9c,d.

**Figure 10:**
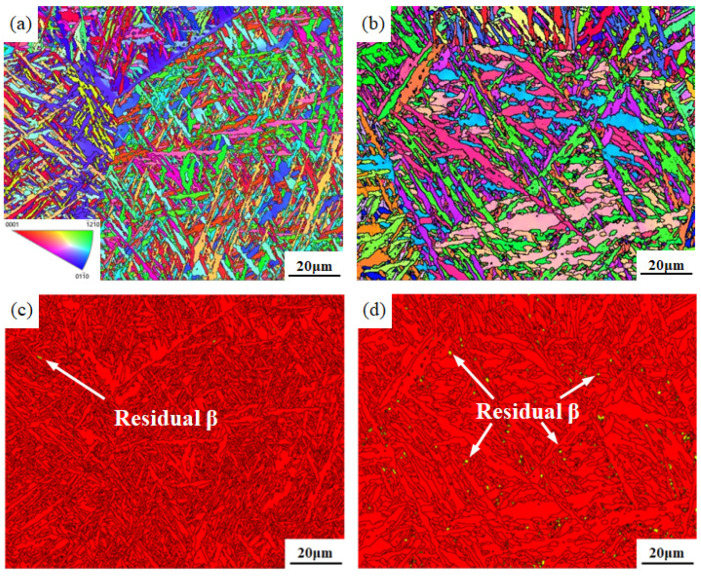
Comparison of the IPF and phase distribution of the welding joints: (**a**,**b**) IPF for flux-cored wire and cable wire welding joints; (**c**,**d**) phase distribution for flux-cored wire and cable wire welding joints.

The IPF diagrams of the fusion zones of the two welded joints show that a large amount of acicular α′ martensite with fine size and a small aspect ratio exists in the fusion zone microstructure of the flux-cored wire welded joint. Acicular α′ martensite with different orientations is interwoven and dispersively distributed, as shown in Figure 10a. In this state, the microstructure can exert a dispersion-strengthened effect [34]. The fusion zone of the flux-cored wire welded joint is composed of an α phase with a close-packed hexagonal structure and very few β-phases with a body-centered cubic structure (accounting for 0.04%), as shown in Figure 10c (red represents the α phase, and yellow represents the β phase). In comparison, the directionality of acicular α′ martensite in the microstructure of the cable wire welded joint is more obvious. Its acicular morphology is more typical and coarse, with a large aspect ratio and size, as shown in Figure 10b. The fusion zone of the cable wire welded joint is also dominated by the α phase, but a small amount of residual β is locally present (accounting for 0.67%), as shown in Figure 10d.

**Figure 11:**
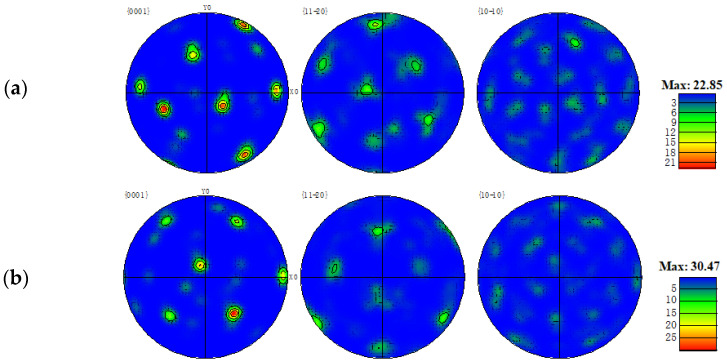
Comparison of textural orientation: (**a**) flux-cored wire welding joint; (**b**) cable wire welding joint.

**Figure 12:**
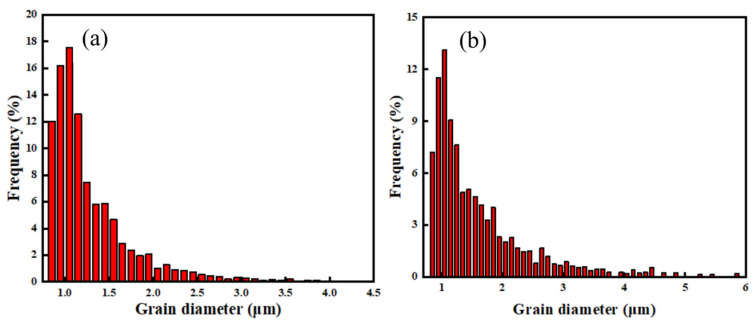
Comparison of grain sizes: (**a**) flux-cored wire welding joint; (**b**) cable wire welding joint.

This microstructural directionality usually indicates stronger textural orientation strength, as reflected by the comparison of the polar figure of the two welded joints in Figure 11. The maximum textural orientation strength of the fusion zone of the flux-cored wire welded joint is 22.85. In contrast, the maximum textural orientation strength of the fusion zone of the cable wire welded joint reaches 30.47, indicating that the microstructure has more obvious anisotropy. The grain size of the fusion zone of the flux-cored wire welded joint is also smaller, as shown in Figure 12. Statistics show that the grain diameter of the fusion zone of the flux-cored wire welded joint tends to lie in the small grain interval, with grains with a diameter of more than 4 μm accounts very little of the fusion zone. In contrast, grains with a diameter of more than 4 μm occupy a greater proportion of the fusion zone of the cable wire welded joint.

### 3.2. Mechanical Property Comparison

**Figure 13:**
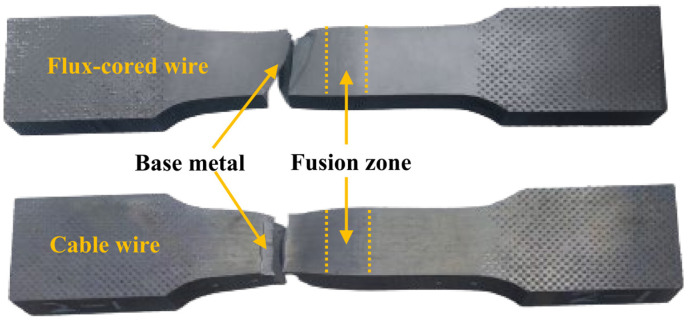
Tensile specimen fracture location of flux-cored wire and cable wire welding joints.

**Figure 14:**
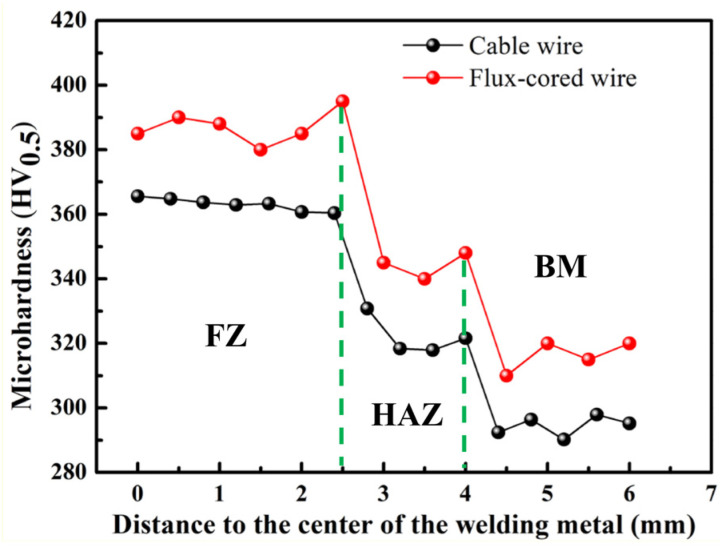
Microhardness comparison of flux-cored wire and cable wire welding joints.

It is known that the differences in the microstructure of welded joints are ultimately reflected in the mechanical properties [35], so the mechanical properties of both welded joints were compared, including tensile properties and impact properties. The exact values are listed in Table 3. In the tensile process of the two welded joints, it was found that the specimen fracture did not occur in the fusion zone (the middle of the tensile specimen) but in the base metal region, as shown in Figure 13. This indicates that the joint strength of the two welded joints is significantly higher than that of the base metal, meaning that the TC4 titanium alloy joints can meet the requirements of engineering applications [36]. However, because the fracture happened in the base metal region, the tensile properties (tensile strength, yield strength, and elongation) and tensile fracture were not analyzed, as such a fracture is only related to the properties of the base metal. Figure 14 shows a comparison of the overall microhardness distribution of the flux-cored wire and cable wire welded joints. The overall distribution pattern occurs in the following order, supporting the tension results: FZ > HAZ > BM.

**Figure 15:**
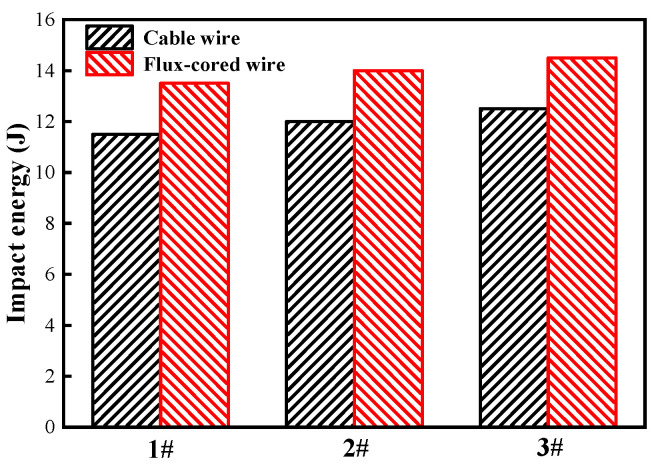
Charpy impact energy comparison histogram of flux-cored wire and cable wire welding joints.

**Figure 16:**
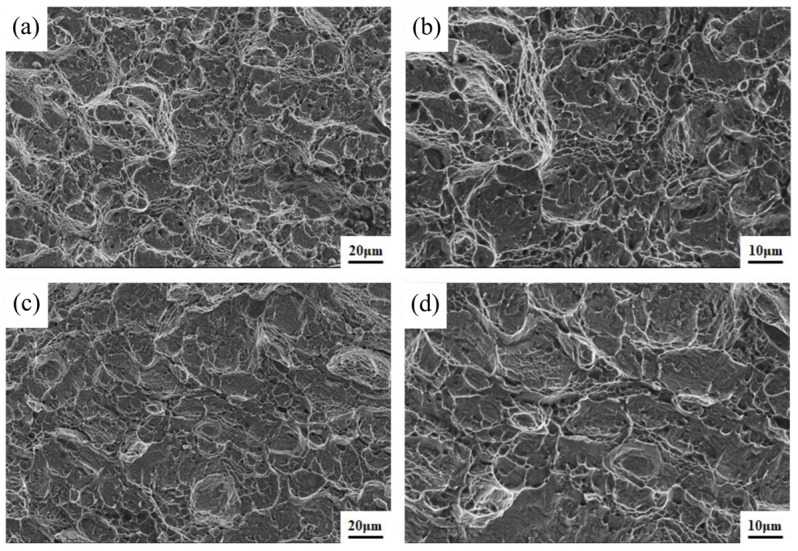
Impact fracture morphology comparison of flux-cored wire and cable wire welding joints: (**a**,**b**) flux-cored wire welding joint; (**c**,**d**) cable wire welding joint.

Impact performance is an essential indicator of the quality of TC4 titanium alloy welded joints. After tensile testing, the results of Charpy impact tests at room temperature were compared. The results show that the average impact energy of flux-cored wire welded joints is 14J, which is 16.7% higher than that of cable wire welded joints (12J). The specific comparison results are shown in Figure 15. Differences in impact performance can are also reflected in the impact fracture morphology, as shown in Figure 16. The impact fracture of the flux-cored wire welded joint shows a ductile fracture feature. In the fracture, a certain number of dimples with large and shallow characteristics can be observed, which may be related to the coarse microstructural state of the fusion zone, as shown in Figure 16a,b. Moreover, the impact fracture of the cable wire welded joint presents a quasi-ductile fracture feature. There are fewer and flatter dimples in the fracture, limiting the effect and preventing crack expansion, as shown in Figure 16c,d. Judging from the fracture morphology of both joints, the impact toughness of flux-cored wire welded joints is better than that of cable wire, which is consistent with the results of the impact energy result presented in Figure 15. The differences in fracture performance are mainly due to the various differences in microstructural characteristics between the flux-cored wire and cable wire welded joints. The size of the acicular α′ martensite phase in the microstructure of the flux-cored wire welded joint is smaller, the distribution of the lath bundle is more dispersed, and the characteristics of the basketweave structure are less obvious. In contrast, a β phase and a large amount of acicular α′ martensite with a large aspect ratio exist in the fusion zone of the cable wire welded joint, providing higher strength [37] but making the fusion zone more brittle and less ductile.

The reason for the differences in the microstructure and mechanical properties between flux-cored and cable wire welded joints may be that flux-cored wire plays a role in improving the stability of the weld, enabling a more uniform and stable transition of the molten droplets [38]. The flux core structure in the wire is more likely to transit the alloying elements to the melt pool in a multielement microalloying manner, playing a metallurgical role and making the alloying elements less prone to element aggregation. This can ensure that the critical alloying elements are more diffuse in the microstructure, promoting the nucleation of the liquid metal in the melt pool, which increases the percentage of large-angle grain boundaries in the microstructure and the resistance to crack extension. In addition, the alloying elements with special features in the flux core are more easily transited to the weld through the metallurgical welding process, leading to the formation of fine carbides to achieve solid solution strengthening of the matrix, effectively enhancing the comprehensive performance and improving the coarse microstructure and anisotropy of the welded joint [39,40]. In contrast, the structural characteristics of cable wire determine the melting process. The relative positions of different strands change continuously during melting, causing the melt pool to rotate in the direction opposite to that of the twisted strands. This promotes the stirring effect of the liquid metal in the melt pool to intensify the flow of the melt pool. Thus, the transfer of heat from the melt pool to the sidewall is accelerated, which positively improves the sidewall depth of fusion, refining the grain and improving the comprehensive mechanical properties of the welded joints [41]. In addition, cable wire, owing to its unique structure, shows a higher efficiency of melt drop transition during the welding process, which can achieve faster welding speed and significantly improve welding efficiency. However, the flux-cored wire generally performs better in welding thick-walled TC4 titanium alloys.

## 4. Conclusions

Welded joints of thick-walled TC4 titanium alloy were obtained using flux-cored wire and cable wire welding separately. The two welded joints were compared in terms of their microstructural characteristics, texture, tensile properties, and impact energy. Based on the characteristics of flux-cored wire and cable wire and the welding principle, the reasons for the differences between the two welded joints were elaborated. The conclusions are as follows:

(1) A coarse β microstructure can be observed in the two welded joints. The basketweave structure consists of a large amount of interwoven acicular α′ martensite, which is dominant in the β microstructure. The β microstructure in the joint welded with flux-cored wire presents with blocky polygonal morphology, whereas the β microstructure in the joint welded with cable wire shows a coarse columnar dendrite morphology with more typical and stronger internal acicular α′ martensite and more obvious directionality.
(2) The strength of the joints welded with flux-cored and cable wire is higher than that of the base metal. During the tensile tests, fractures occurred in the base metal region, indicating that the welded joints can meet the requirements of engineering applications. The impact energy of the flux-cored wire welded joint at room temperature is higher than that of the cable wire joint by 16.7%, and the fracture morphology shows toughness fracture.
(3) As a filler material, flux-cored wire achieves better performance in terms of the microstructure and mechanical properties when using a laser to weld TC4 titanium alloy. This is mainly because the flux-cored structure transits the alloying elements into the molten pool in the form of multielement microalloying. It also plays a metallurgical role in enhancing the comprehensive performance and improving the coarse microstructure and anisotropy in welding thick-walled TC4 titanium alloy.

## Figures and Tables

**Table 1:** Chemical composition of the base metal and filler wires (mass fraction, %).

Material	Al	V	Fe	C	N	H	O	Ti
Base material	6.30	4.11	0.018	0.024	0.007	0.001	0.14	Balance
Flux-cored wire	6.10	4.15	0.040	0.012	0.006	0.001	0.02	Balance
Cable wire	6.20	3.92	0.020	0.028	0.005	0.001	0.10	Balance

**Table 2:** Welding parameters.

Process Parameter	Flux-Cored Wire Welding	Cable Wire Welding
Laser power (W)	3000	4000
Welding speed (mm/min)	0.65	0.42
Wire feeding rate (mm/min)	1.5	3.5
Defocusing distance (mm)	20	20
Oscillation pattern	Circle	Circle
Oscillation frequency (Hz)	100	100
Oscillation diameter (mm)	2	2
Shielding gas	Ar	Ar
Gas flow rate (L/min)	35	35

**Table 3:** Mechanical properties of the welding joints.

Mechanical Property	Flux-Cored Wire Welding	Cable Wire Welding
Tensile strength (MPa)	925	941
Elongation (%)	12.5	11
Fracture location for tension	BM	BM
Charpy impact energy (J)	14	12

## Data Availability

No new data were created or analyzed in this study. Data sharing is not applicable to this article.
